# Poster Session II - A300 THE CLINICAL COURSE AND MAGNETIC RESONANCE IMAGING FINDINGS IN ISOLATED PERIANAL CROHN’S DISEASE

**DOI:** 10.1093/jcag/gwaf042.300

**Published:** 2026-02-13

**Authors:** J Chambers, L Jackman, P Belesiotis, M Cino, A N Sasson, A Chadi, P Tandon

**Affiliations:** University of Toronto, Toronto, ON, Canada; University of Toronto, Toronto, ON, Canada; University of Toronto, Toronto, ON, Canada; University Health Network, Toronto, ON, Canada; Gastroenterology, University of Toronto, Toronto, ON, Canada; University Health Network, Toronto, ON, Canada; University Health Network, Toronto, ON, Canada

## Abstract

**Background:**

Crohn’s disease (CD) can manifest with perianal symptoms, including abscess/fistulae, which result in significant morbidity. These perianal manifestations of disease can occur despite a complete absence of luminal CD, thus being termed Isolated Perianal CD (IpCD). The natural history and radiographic description of Isolated perianal (IpCD) remains largely uncharacterized.

**Aims:**

We report a case series of patients diagnosed with IpCD at a tertiary care center, where the aim is to characterize the incidence of IpCD, the delay in CD diagnosis, and the typical imaging findings/clinical course of the ipCD phenotype.

**Methods:**

We conducted a retrospective chart review on patients with ipCD and applied descriptive statistics to characterize the incidence, clinical course, and MRI findings of the disease.

**Results:**

We identified 20 patients with IpCD between 2017-25 for an incidence of 1.5%. All patients had normal initial colonoscopies and small bowel imaging, and all patients were initially managed by general surgery. The mean time from symptom onset to diagnosis was 3.4 ± 2.9 years. During this period 13/20 (65%) required antibiotics, and 16/20 (80%) required surgical management in addition to 14/20 (70%) presenting to the emergency department and 13/20 (65%) requiring hospitalization. Only 7/20 (35%) of patients were started on anti-TNF biologics within the mean follow-up period of 3.2 ± 2.4 years. We also find 1.14 vs. 0.36 ED visits/patient-years and 0.70 vs. 0.16 hospitalizations/patient-years in the pre vs. post anti-TNF treated groups respectively. 7/20 (35%) of patients reached clinical remission in the study and only 1 developed microscopic findings of luminal CD. For imaging findings, 19/20 patients (95%) had at ≥ 1 fistula of which 18/19 (95%) were complex with a median maximal St. James classification of 4 (IQR=2). Fistula types by Parks classification demonstrates a wide variety, where out of 17 classifiable patients 5 (29%) had intersphincteric, 5 (29%) had mixed subtype, and 6 (35%) had transsphincteric. Additionally, we find that 13/20 (65%) had concurrent abscess formation.

**Conclusions:**

Ultimately, we found that there was a significant delay between the onset of perianal symptoms and diagnosis of IpCD, with this delay time characterized by complex perianal fistulas requiring frequent surgical interventions, need for antibiotics, healthcare utilization, and overall morbidity. Earlier identification of these patients and their starting on medical therapy, including anti-TNF class biologics, may improve outcomes and further work should focus on the development of prediction models to identify patients at high risk of having IpCD.

**Funding Agencies:**

None

